# The narrow subspine space and relatively large labrum are radiographic features of subspine impingement: a case-control study

**DOI:** 10.1186/s12891-022-05947-w

**Published:** 2022-11-19

**Authors:** Rongge Liu, Yuqing Zhao, Yan Xu, Huishu Yuan

**Affiliations:** 1 Department of Sports Medicine, Peking University Third Hospital, 49 North Garden Road, Haidian District, 100191 Beijing, China; 2 Department of Radiology, Peking University Third Hospital, 49 North Garden Road, Haidian District, 100191 Beijing, China

**Keywords:** Arthroscopy, Subspine impingement, Imaging features, Hip joint

## Abstract

**Background:**

Subspine impingement is considered a source of residual hip symptoms after primary hip arthroscopy, and the role of the subspine space and soft tissue is not clear. The purpose of this study was to analyze the relationship between the subspine space and labrum size in subspine impingement patients.

**Methods:**

We performed a retrospective study of patients with femoroacetabular impingement between July 2016 and July 2020. Sixteen patients without hip symptom relief after primary hip arthroscopic treatment of femoroacetabular impingement and undergoing revision surgery for anterior inferior iliac spine compression were included as the study group. Forty-eight matched patients who underwent only primary surgery and whose hip discomfort was relieved without a diagnosis of subspine impingement were included as the control group. The patients’ preoperative computerized tomography data were reviewed, and the anterior inferior iliac spine dimensions and the size of the subspine space were measured. The size of the labrum at the 11:30, 1:30, and 3 o’clock positions was measured with the use of magnetic resonance imaging. The ratio of the subspine space to the labrum was also calculated.

**Results:**

There was no significant difference in anterior inferior iliac spine dimensions between these two groups (*p* > 0.05). A relatively narrow subspine space was found in the study group, especially in the direction of the anterior inferior iliac spine. Compared with the control group, subspine impingement patients were identified with larger labrums at 11:30 (8.20 ± 1.95 mm vs. 6.81 ± 0.50 mm, *p* = 0.016), 1:30 (7.83 ± 1.61 mm and 6.25 ± 0.78 mm, *p* = 0.001) and 3:00 (9.50 ± 1.73 mm vs. 7.48 ± 0.99 mm, *p* = 0.001). A relative mismatch between the subspine space and the labrum was also identified in the study group. The ratios of the labrum width to the subspine area were significantly larger in the study group than in the control group.

**Conclusion:**

This study reported potential additional criteria for subspine impingement—a large labrum and a relatively narrow subspine space—instead of abnormal anterior inferior iliac spine dimensions. For those with a large labrum and narrow subspine space, the diagnosis of subspine impingement should be carefully made, and arthroscopic anterior inferior iliac spine decompression may be important.

## Background

Femoroacetabular impingement (FAI) has been widely accepted as a potential source of hip pain and limited range of motion (ROM) in young adults, but there are many FAI patients who undergo hip surgeries for FAI and still have residual hip symptoms [1–3]. Recently, extra-articular hip impingement, such as subspine impingement (SSI), has increasingly been recognized as another important source of hip symptoms in young people after primary hip arthroscopy procedures, especially residual hip pain in straight flexion [4–10]. Statistically significant improvement in the clinical outcomes of arthroscopic anterior inferior iliac spine (AIIS) decompression has been reported by many studies [11–15]. For patients with SSI, arthroscopic treatment of AIIS decompression is the critical treatment to achieve better clinical outcomes, while for those without SSI, unnecessary decompression of the AIIS can cause potential unexpected damage. Therefore, accurate presurgery diagnosis of SSI is important for the management of hip discomfort.

Imaging evaluation is significant in the differential diagnosis because the clinical findings of both FAI and SSI, including symptoms and physical examination findings, are similar [16]. Many studies have reported that there is a certain relationship between the morphology of the AIIS and clinical symptoms [17–21]. As initially described by Hetsroni et al., the morphology of the AIIS could be classified into three types based on three-dimensional computerized tomography (3D-CT), and a prolonged AIIS was associated with decreased ROM [19]. Recently, the correlation of SSI symptoms and the morphology of the AIIS has been questioned.[13, 22, 23] Bernardo et al. reported that 23.7% of symptomatic FAI patients were identified as having SSI, and more than half of them (52.2%) were associated with type I AIIS instead of type II or type III [10]. Samim et al. reported different magnetic resonance imaging (MRI) features beyond the morphology of the AIIS, such as distal femoral cam deformity and signs of the impingement of the distal femoral neck, in patients with or without SSI [20]. Most of the existing studies were focused on the morphology of the AIIS that caused insufficient space for the labrum in patients with SSI. However, the difference in subspine space between the SSI group and the non-SSI group was not clear. On the other hand, a larger labrum may cause relatively insufficient space and cause potential labrum damage in terminal hip flexion, and few studies have focused on the relationship between the subspine space and labrum.

The purpose of this study was to analyze the relationship between the subspine space and labrum size in patients with or without SSI. Our hypothesis is that one of the distinguishing imaging features of SSI patients is the mismatch between the narrow subspine space and the large labrum instead of the abnormal size of the AIIS.

## Methods

We reviewed our database and selected patients who underwent hip arthroscopic procedures performed by our senior author because of symptomatic FAI between July 2016 and July 2020. Our study was a retrospective review and was approved by the institutional review board according to the Helsinki recommendation.

### Study Population

Patients were included in this study if they (1) had a diagnosis of symptomatic FAI for which nonoperative treatment failed for at least 6 months, (2) underwent hip arthroscopy surgery at our institution and performed by our senior author (clinical surgeon in sports medicine, with more than 15 years of experience in hip arthroscopy), and (3) had preoperative 3D-CT examinations of the hip joint at our institution. Our exclusion criteria were patients (1) with radiographic evidence of osteoarthritis (Tonnis Grade > 1); (2) with a history of open hip surgery; and (3) with rheumatoid arthritis, avascular necrosis of the femoral head, Legg-Calve-Perthes disease, slipped capital femoral epiphysis, or hip dysplasia (lateral center-edge angle (LCEA) < 20°).

As a comorbid condition of FAI and a reason for revision surgeries for FAI patients, there are few gold standard diagnostic tests for the differential diagnosis of SSI. Furthermore, the correlation of SSI symptoms and the morphology of the AIIS has been questioned [13, 22, 23]. Thus, to identify patients with SSI and differentiate SSI from FAI, comprehensive diagnostic criteria based on clinical history, hip symptoms, and physical examination and radiographic or sonographic evaluation findings were used. In the present study, patients were included in the study group (SSI group) if they (1) still had residual hip symptoms after primary surgery in which no AIIS decompression was performed and (2) benefited from secondary hip arthroscopic AIIS decompression, with or without ultrasound-guided AIIS injection before their revision surgeries. Patients were included in the control group (non-SSI group) if they underwent only one hip arthroscopic ipsilateral surgery for FAI, in which no AIIS decompression was performed, and achieved significant improvement in clinical outcomes. The study group was matched in a 1:3 ratio with the control group in terms of age, sex, and body mass index (BMI).

### Diagnosis

The diagnosis of FAI or SSI was made by our senior author (clinical surgeon with more than 15 years of experience in sports medicine). We collected information on the medical history, symptoms, physical examination findings, and hip function. The range of motion (ROM) was also measured for diagnosis. Patients with a large alpha angle (alpha angle > 55°) and acetabular retroversion were considered to have FAI syndrome.

Patients were considered positive for SSI if they underwent secondary surgery in which AIIS decompression was performed and achieved a significant improvement in clinical symptoms. Patients with typical symptoms of SSI, such as anterior hip pain and hip pain over the AIIS in deep hip flexion, were more likely to have SSI syndrome. Pain during passive hip flexion with the hip in neutral rotation, as well as limited ROM and tenderness over the AIIS, was meaningful for the diagnosis of SSI, which is known as the subspine impingement test [14]. Ultrasound-guided injection in the AIIS was performed in most suspected patients before their secondary hip arthroscopic treatment, and positive results supported the diagnosis of SSI. Arthroscopic evidence of SSI syndrome included labrum damage at 2–3 o’clock, capsule edema, an injured straight head of the rectus femoris, and hypertrophic soft tissue in the subspine space.

### Surgical technique

All hip arthroscopy surgeries were performed by our senior author (clinical surgeon with more than 15 years of experience in sports medicine) in the standard supine position on a hip distraction table. Standard portals, such as the lateral portal and mid-lateral portal, were used in our surgery. In the present study, cam or pincer deformities were treated by arthroscopic osteochondroplasty. All loose bodies were removed by lavage or a grasper, and labrum tears were repaired using the multisuture anchor technique. Labrum reconstruction was also performed if the labrum repair was difficult to perform. Capsular plication was performed arthroscopically for patients with borderline developmental dysplasia of the hip whose LCEA was between 20° and 25°. In revision cases, AIIS decompression was performed in patients who were diagnosed with SSI syndrome using a 4-mm arthroscopic bur.

### Radiographic evaluation

Our first author (musculoskeletal fellowship-trained radiologist with more than 8 years of experience) reviewed the radiographic data and assessed the CT and MRI before the first hip arthroscopy. In our study, the size and location of the AIIS, the subspine space and the labrum size were measured. CT scans were used to describe the dimensional sizes and locations of the AIIS and the subspine space, while MRI scans were used to evaluate the labrum width.

To quantify the dimensional size of the AIIS, we measured the length, width and height of the AIIS, as initially described by Eyal et al. [17] In addition, we measured the AIIS version, including two angles -- the first angle formed between the AIIS mid-axis line and the ilium mid-axis line (angle with ilium) and the second angle formed between the AIIS mid-axis line and a plumb line (angle with plumb line), which were also described by Eyal et al. [17] In our study, the AIIS version and the width of the AIIS were measured in the axial view, and the length and height of the AIIS were measured in the oblique sagittal view, which is not perpendicular but oblique through the axis of the AIIS.

**Fig. 1:**
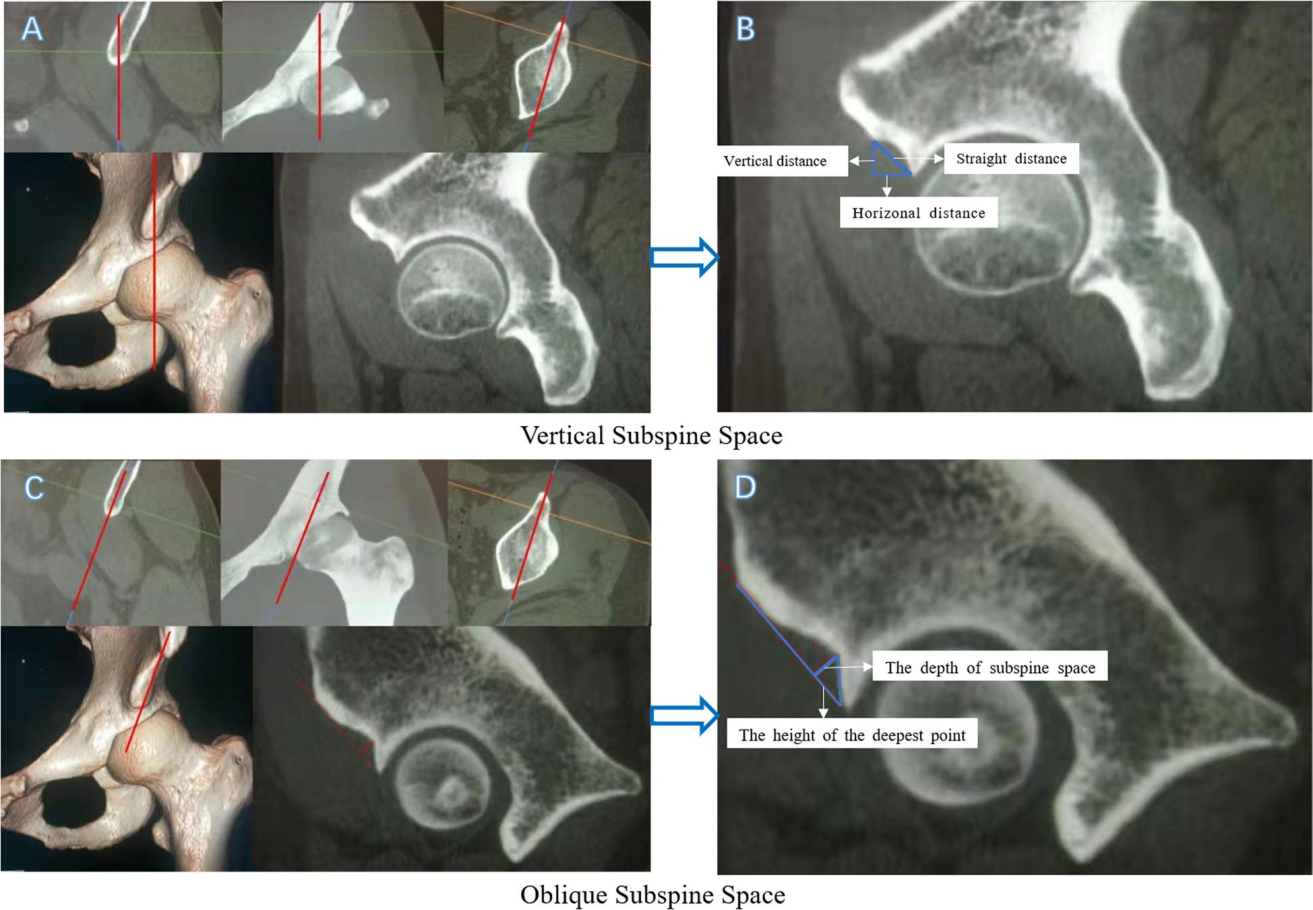
Measurement of the subspine space in the vertical and oblique directions

Legend: (A) The sagittal plane we selected for the measurement of the vertical subspine space. (B) The details of the measurement of the vertical subspine space: the vertical, horizontal, and straight distances from the AIIS to the acetabular rim. (C) The oblique sagittal plane we selected for the measurement of the oblique subspine space. (D) The details of the measurement of the oblique subspine space: the depth of the subspine space and the height of the deepest point.

We evaluated the subspine space in the perpendicular plane as well as in its natural direction. As shown in Fig. 1, we marked the most anterior point of the AIIS in the sagittal view on 3D-CT and then measured the vertical, horizontal, and straight distances from the most anterior point of the AIIS to the acetabular rim, which was named the “vertical subspine space” in the present study. However, as the natural axis of the AIIS was not vertical to the horizontal plane, we therefore evaluated the “oblique subspine space” in the oblique sagittal view, which was parallel to the natural plane of the AIIS and was previously used in the measurement of the length and height of the AIIS. In this oblique sagittal view, the deepest point of the subspine space was marked, and a line from the most anterior point of the AIIS to the acetabular rim was created. As shown in Fig. 1, the marked point, the created line, and the acetabular rim formed a right triangle. The two legs of this right triangle showed the depth of the subspine space and the height of the deepest point, and the area of this right triangle was calculated, which was named the “oblique subspine area”.

**Fig. 2:**
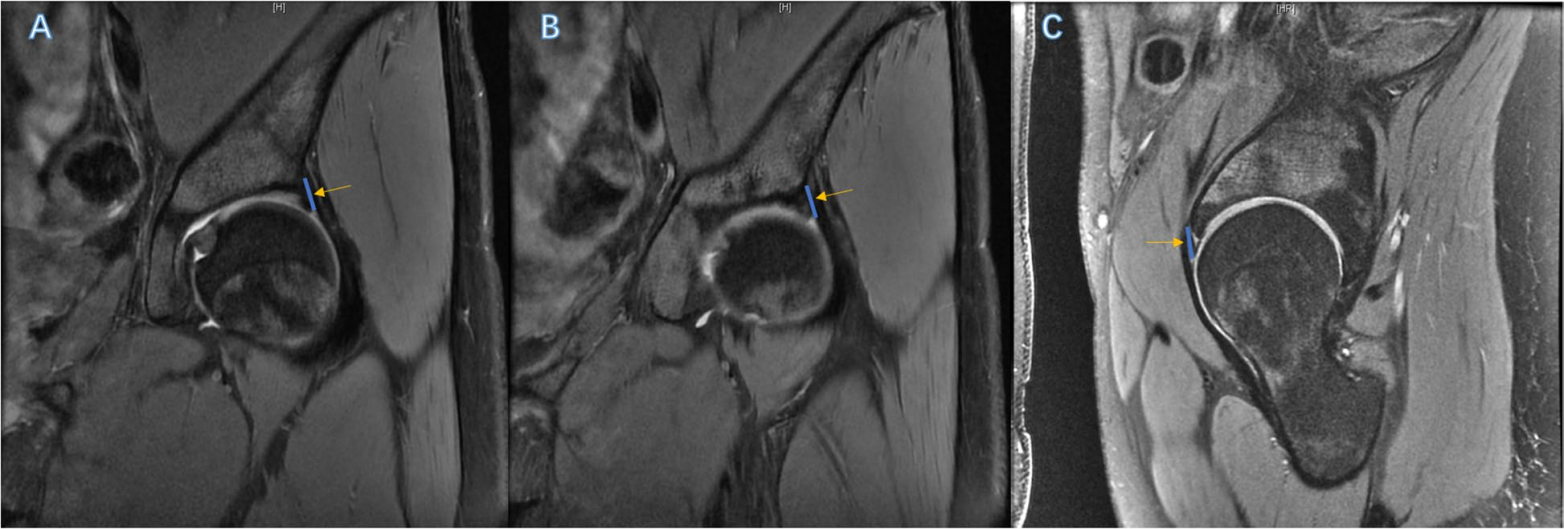
The measurement of the labral width in three standard positions in MRI scans. Legend: All three figures show the left hip. **A** The insertion of the indirect rectus (11:30) (**B**) The half-point of the indirect rectus insertion and the psoas U (1:30). **C** The psoas U (3:00).

We measured the width of the labrum at 11:30, 1:30, and 3:00 in MRI scans. Three standardized locations of the anterior to superior labrum were used for the measurements, as described by several studies [24, 25]. The length of the labrum was measured at the 11:30 clock-face position on a coronal proton density (PD) sequence, in which we used the posterior border of the indirect head of the rectus femoris tendon as the landmark; at the 3-o’clock position on an axial oblique PD sequence at the psoas U, where the iliopsoas tendon passes anterior to the labrum; and at the 1:30 clock-face position, a half-point between the 11:30 and 3:00 positions on a sagittal fat-suppressed PD image. (Fig. 2) The ratio of the labral size to the subspine area, including the vertical subspine area and the oblique subspine area, was calculated to assess the relationship between the subspine space and the labrum.

#### Statistical analysis

The matching process of the SSI group and non-SSI group was performed by the MatchIt package in R software (Version 4.0.3; R Project for Statistical Computing), in which the propensity score matching (PSM) method was used. The nearest method was utilized in the matching process, where patients in the control group could be matched only once to those in the study group.

Descriptive statistics, such as the means and standard deviations, were calculated and listed as continuous variables. The paired t test (two-tailed) was used to evaluate any statistically significant differences between the SSI group and the non-SSI group. Receiver operating characteristic curve (ROC) analysis was also performed to assess the diagnostic values of the ratio of the labral width to the subspine area, and the areas under the ROC curves (AUCs) were calculated. The 95% confidence intervals (95% CIs) of the AUCs was also calculated. In our present study, statistical significance was set at *P* < 0.05, and SPSS 25.0 software (SPSS Inc., Chicago, IL, USA) was used for statistical analysis.

## Results

### Study participants

In this study, a total of 64 patients were included. In the SSI group, 16 patients were identified. Forty-eight patients were matched in the non-SSI group. (Table 1) Of these 64 patients, the mean age (± standard deviation) was 39.42 (± 9.83) years; 42 of the 64 patients (65.7%) were female, and 18 patients underwent right hip arthroscopy surgeries. The mean BMI of these included patients was 21.68 (± 3.32) kg/m2, with a mean height of 165.19 (± 8.12) cm and a mean weight of 59.32 (± 10.76) kg. There was no significant difference in age, sex, BMI, α angle, LCEA, or neck-shaft angle between the SSI group and the non-SSI group. There were 20 patients with type I AIIS in the non-SSI group, and 13 patients in the SSI group were identified with type II AIIS. Seven of 16 patients in the SSI group underwent ultrasound-guided AIIS injection after the primary surgery and before the secondary arthroscopy, and all seven patients experienced symptom relief after injection.

**Table 1:** Patient Characteristics and Radiographic Parameters

	Total (*n* = 64)	SSI group (*n* = 16)	Non-SSI group (*n* = 48)	*P* value
Age (years)	39.42 (± 9.83)	38.50(± 6.48)	39.73(± 10.76)	0.587
Sex				
Male	22	6	16	0.769
Female	42	10	32	
Side				
Right	42	10	32	0.769
Left	22	6	16	
BMI (kg/m2)	21.68(± 3.32)	21.65(± 3.52)	21.70(± 3.29)	0.958
α Angle	60.08(± 5.76)	59.67(± 6.01)	60.22(± 5.73)	0.745
LCEA	32.90(± 5.54)	33.11(± 5.77)	32.83 (± 5.53)	0.863
Neck-shaft angle	131.41(± 6.17)	129.56(± 5.16)	132.03 (± 6.41)	0.166
AIIS type				
I	22	2	20	**0.009**
II	40	13	27	**0.009**
III	2	1	1	**0.009**

Unless otherwise specified, data are presented as the mean ± standard deviation or the numbers of hips, with percentages shown in parentheses. Distances are given in millimeters, and angles are given in degrees

*SSI* Subspine impingement, *BMI* Body mass index, *LCEA* Lateral center-edge angle, *AIIS* Anterior inferior iliac spine

### Morphology of the AIIS

The mean width, length, height, angle with the ilium, and angle with plumb line of the AIIS were 11.01(± 1.30) mm, 25.27(± 2.61) mm, 11.70(± 5.22) mm, 11.64(± 3.62)° and 16.86(± 4.73)° in the non-SSI group and 11.04(± 1.33) mm, 26.56(± 5.39) mm, 10.67(± 6.03) mm, 14.48(± 7.09)° and 14.64(± 6.11)° in the SSI group, respectively. In the SSI group, the mean vertical, horizontal and straight distances from the AIIS to the acetabular rim were 22.57(± 4.56) mm, 16.74(± 4.26) mm and 14.43(± 3.56) mm, respectively, and those of the non-SSI group were 24.47 (± 3.22) mm, 20.49 (± 8.16) mm and 15.98 (± 2.11) mm, respectively. The mean area of the triangle formed by the vertical distance and the horizontal distance, also named the vertical subspine area, was 122.53(± 47.34) mm2 in the SSI group and 160.96(± 51.06) mm2 in the control group. No significant difference was identified in AIIS measures between groups in our study. (Table 2)

**Table 2:** Measurements of the AIIS and subspine space

Measurement	SSI group	Non-SSI group	*P* value
Width	11.04(± 1.33)	11.01(± 1.30)	0.942
Length	26.56(± 5.39)	25.27(± 2.61)	0.350
Height	10.67(± 6.03)	11.70(± 5.22)	0.289
Angle with ilium	14.48(± 7.09)	11.64(± 3.62)	0.188
Angle with the plumb line	14.64(± 6.11)	16.86(± 4.73)	0.233
Vertical distance	22.57(± 4.56)	24.47(± 3.22)	0.278
Horizonal distance	16.74(± 4.26)	20.49(± 8.16)	0.163
Straight distance	14.43(± 3.56)	15.98(± 2.11)	0.169
Vertical subspine area	122.53(± 47.34)	160.96(± 51.06)	0.075
The depth of subspine space	2.38(± 1.24)	3.73(± 1.19)	**0.015**
The height of the deepest point	9.40(± 1.74)	9.08(± 2.64)	0.692
Oblique subspine area	11.56(± 7.85)	21.05(± 8.27)	**0.006**

All values are given as the mean ± standard deviation. Distances are given in millimeters, and angles are given in degrees

*SSI* Subspine impingement

### The size of the subspine space

The mean depth of the subspine space and the average height of the deepest point were 2.38(± 1.24) mm and 9.40(± 1.74) mm in the SSI group, respectively, with a mean oblique subspine area of 11.56(± 7.85) mm2, which was a right triangle formed by the depth of the subspine space and the height of the deepest point. In the control group, the mean depth of the subspine space was 3.73(± 1.19) mm, the mean height of the deepest point was 9.08(± 2.64) mm, and the mean oblique subspine area was 21.05(± 8.27) mm2. Two patients (one patient in the SSI group and another in the control group) were identified with type III AIIS, and their depth of the subspine space, as well as oblique subspine area, were considered zero. SSI patients had a smaller subspine space in both the vertical direction and the oblique direction. There were significant differences in the depth of subspine space (*p* = 0.015) and oblique subspine area (*p* = 0.006). (Table 2)

### Labrum size

**Fig. 3:**
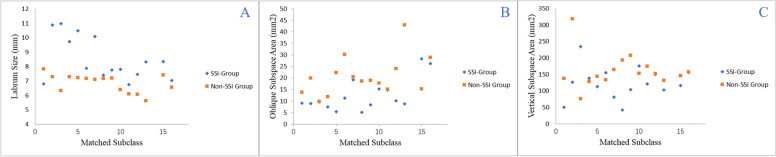
Comparison of the labrum size and subspine area. Legend: **A** Comparison of the average labrum width between the SSI group and the non-SSI group. **B** Comparison of the vertical subspine area. **C** Comparison of the oblique subspine area.

One patient in the control group and one patient in the study group lacked measurable MRI data. The missing data of the patients in the control group were replaced by the mean values of the other two patients in the same group. The mean width of the labrum at 11:30, 1:30 and 3:00 was 8.20 (± 1.95) mm, 7.83 (± 1.61) mm and 9.50 (± 1.73) mm in the SSI group and 6.81 (± 0.50) mm, 6.25 (± 0.78) mm and 7.48 (± 0.99) mm in the matched control group, respectively. (Table 3) The sizes of the labrum at all three positions in the control group were significantly smaller than those in the SSI group (Fig. 3).

**Table 3:** The size of the labrum

Position	SSI group		Non-SSI group	*P* value
11:30		8.20 (± 1.95)	6.81 (± 0.50)	**0.016**
1:30		7.83 (± 1.61)	6.25 (± 0.78)	**0.001**
3:00		9.50 (± 1.73)	7.48 (± 0.99)	**0.001**

All values are given as the mean ± standard deviation. Distances are given in millimeters

*SSI *Subspine impingement

### The relationship between the subspine space and the labrum

The ratios of the labrum width to the oblique subspine area at 11:30, 1:30 and 3:00 were 0.83 (± 0.44) mm-1, 0.81 (± 0.46) mm-1 and 0.98 (± 0.52) mm-1 in the SSI group and 0.50 (± 0.17) mm-1, 0.47 (± 0.19) mm-1 and 0.56 (± 0.22) mm-1 in the non-SSI group, respectively. (Table 4) The ratios of the labrum width to the vertical subspine area at 11:30, 1:30 and 3:00 were 0.08 (± 0.03) mm-1, 0.07 (± 0.04) mm-1 and 0.09 (± 0.05) mm-1 in the SSI group and 0.06 (± 0.04) mm-1, 0.06 (± 0.04) mm-1 and 0.07 (± 0.05) mm-1 in the non-SSI group, respectively. As shown in Table 4, there was no significant difference between these two groups in any ratios of the vertical subspine area, and patients with SSI had smaller ratios of the oblique subspine area.

**Fig. 4:**
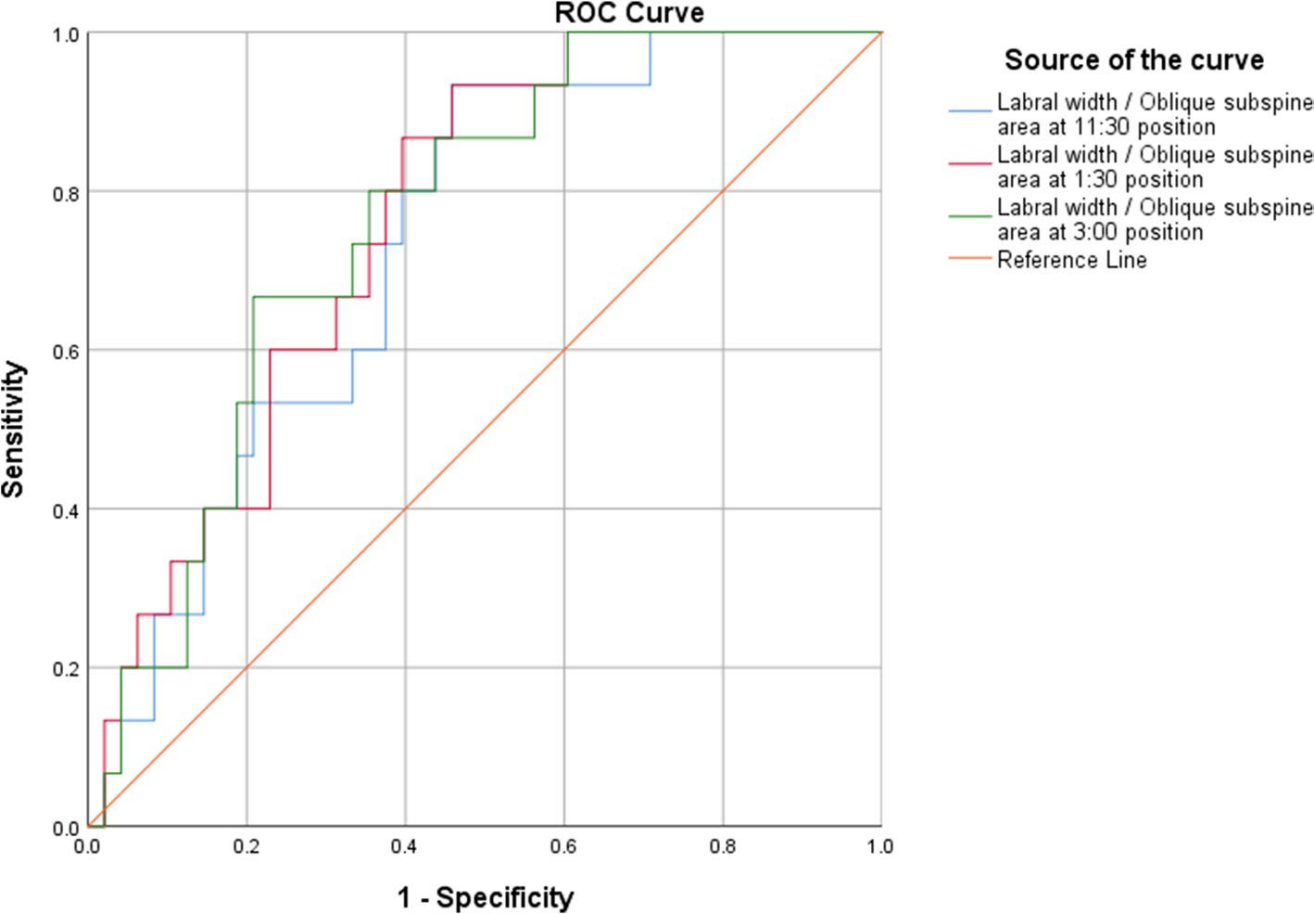
The ROC curves of the ratios of the labral width to the oblique subspine area. Legend: This figure shows the ROC curve analysis of the ratio of the labral width to the oblique subspine area at three different anatomical sites

The ROC curves of the ratios of the labral width to the oblique subspine area are shown in Fig. 4, and the AUCs of these ratios were 0.733 at the 11:30 position (95% CI: 0.604–0.862, *p* = 0.007), 0.761 at the 1:30 position (95% CI: 0.638–0.884, *p* = 0.002) and 0.761 at the 3:00 position (95% CI: 0.640–0.882, *p* = 0.002). The optimal cutoff values of these ratios were 0.046 at the 11:30 position (sensitivity = 0.93), 0.041 at the 1:30 position (sensitivity = 0.93) and 0.068 at the 3:00 position (sensitivity = 0.67).

**Table 4:** The ratios of the labrum widths to the subspine space areas at three positions

Position	SSI group	Non-SSI group	*P* value
Labrum width/vertical subspine area			
11:30	0.08 (± 0.03)	0.06 (± 0.04)	0.336
1:30	0.07 (± 0.04)	0.06 (± 0.04)	0.245
3:00	0.09 (± 0.05)	0.07(± 0.05)	0.289
Labrum width/oblique subspine area			
11:30	0.83 (± 0.44)	0.50 (± 0.17)	**0.010**
1:30	0.81 (± 0.46)	0.47 (± 0.19)	**0.007**
3:00	0.98 (± 0.52)	0.56 (± 0.22)	**0.004**

All values are given as the mean ± standard deviation. Distances are given in millimeters

*SSI* Subspine impingement

## Discussion

The most important findings in this study were the potentially relative mismatch between the overlarge labrum and the relatively narrow subspine space in SSI patients. SSI patients had a larger labrum than non-SSI patients in all three positions. Additionally, we found that the oblique subspine space of SSI patients, which is not vertical to the horizontal direction but along the natural axis of the AIIS, was smaller than that of patients without SSI.

SSI was identified as abnormal contact between the a prolonged AIIS and the distal femoral neck, as reported by Reith et al. [26] Hetsroni et al. reported a classification system of AIIS morphology and found that a prolonged AIIS was associated with limited ROM [19]. Many studies have reported a correlation between the a prolonged AIIS and SSI symptoms [18, 19, 26]. We measured the morphology of the AIIS, including the width, length, height, angle with the ilium, angle with the plumb line, vertical subspine space and oblique subspine space, and found that SSI patients had a narrower subspine space, especially along the natural plane of the AIIS, instead of other morphological factors. The AIIS was not vertical but oblique, and hyperplasia of the AIIS for various reasons may not follow the vertical direction but may follow its natural anatomical direction. Therefore, the oblique subspine area may be more helpful and sensitive for evaluating hyperplasia of the AIIS.

Recently, studies showed that abnormal morphology of the AIIS was not completely related to hip symptoms [13, 22, 23]. In type II AIIS, in which the AIIS sits at the level of the acetabular rim, the potential impingement of soft tissue between the distal femoral neck and the AIIS, which causes a narrow subspine space, was considered the reason for hip symptoms [19]. Amer et al. reported a case of an SSI patient with a hyperemic anterior labrum and prolonged AIIS [27]. The findings from these studies suggest that the bony structure cannot fully explain the mechanism of SSI, especially in patients without type III AIIS, and impingement may occur between the bony structure and the labrum. We measured the length of the labrum in three positions—11:30, 1:30 and 3 o’clock—and SSI patients were found to have a larger labrum in all three positions. Onur et al. reported that the AIIS is located between 1:00–1:30 and 2:00–2:30 above the acetabular rim.[28]. The labrum in these two positions was below the AIIS, which suggests that not only the bony structure but also the soft tissue, such as the anterosuperior labrum, is important in SSI. In terminal hip flexion, the anterosuperior labrum, which is below the AIIS and located in the subspine space, is more likely to be compressed. Soft-tissue and synovial edema in SSI patients have also been noted by clinical surgeons, and the findings of these studies also support our findings [22].

In this study, the ratios of the subspine area to the length of the labrum were calculated to demonstrate the morphological discrepancy between them. We noticed a larger ratio in the SSI group than in the non-SSI group, not only in the oblique direction but also in the vertical direction. The result indicates a larger labrum and narrower subspine space in SSI patients. As a comorbid factor of FAI, because of the cam or pincer deformity, SSI may not cause direct contact between the AIIS and the bony femoral neck but may make the soft tissue in the subspine space more sensitive to compression, especially during flexion and internal rotation. Kobayashi et al. reported a significantly increased rate of postoperative AIIS impingement by computer simulation analysis based on 3D-CT, which may support that the presence of cam and pincer deformities reduces the occurrence of SSI, and after the removal of cam and pincer deformities, the AIIS is more likely to contact the femoral neck, which supports our findings [29]. One potential explanation is that the narrow subspine space and large anterosuperior labrum made the soft tissue in the subspine space more likely to be compressed in extreme hip flexion in some sports activities and caused clinical symptoms, such as pain and limited ROM.

Many studies have focused on the imaging evaluation of SSI [17–21, 26]. Hetsroni et al. reported limited ROM in patients with a prolonged AIIS [19]. Amar et al. used ultrasound examination to evaluate the morphology of the AIIS and reported great diagnostic value of this technique [18]. Sanim et al. reviewed the MRI features of the SSI group and reported abnormalities in the boney structure and soft tissue, such as distal cam and edema of the distal femoral neck [20]. They did not focus on the area of the subspine space, especially in the oblique direction, or on anterosuperior labrum size. These previous studies required comprehensive diagnostic criteria for SSI, which included a combination of clinical history, hip symptoms, and physical examination and radiological or sonographic examination findings. In this study, we selected the comprehensive criteria for the differential diagnosis of SSI, in which ultrasound-guided AIIS injection and pain relief after secondary arthroscopic AIIS decompression may make the diagnosis of the study group more reasonable.

The clinical physician should pay more attention to the relative correlation of the subspine space and soft tissue, such as the labrum. The relative mismatch between these two structures could be an additional diagnostic criterion for SSI. For those with a large labrum and narrow subspine space, the evaluation of SSI may be important, and AIIS decompression in the primary surgery may be helpful.

Our study has several limitations that must be noted. First, the sample size was too small. We included 64 patients in our study. Because of the sample size, some potential influences of a large labrum and a narrow subspine space were not analyzed. Second, we selected patients who underwent AIIS decompression in their revision surgeries instead of primary surgeries as the SSI group. Because SSI is a type of extra-articular hip impingement and often coexists with FAI with similar symptoms and physical examination findings, patients with a confirmed diagnosis of SSI but without cam or pincer deformities were rare. Further studies may collect data from patients who underwent arthroscopic AIIS decompression without concurrent treatment of FAI as a study group and measure their subspine space. Third, because of the lack of an effective method to assess the size of the hip capsule under the AIIS, we did not evaluate the relationship between the capsule and the subspine space. Further studies may develop a more reasonable soft tissue measurement method to analyze this relationship.

## Conclusion

This study showed the mismatch between a narrow subspine space and a relatively large labrum in SSI patients compared with a matched control group. The mismatch between the larger labrum and the narrow subspine space could be an additional radiographic criterion for the diagnosis of SSI.

## Data Availability

All relevant data supporting the conclusions are included within the article and tables. The datasets used and/or analyzed during the current study are available from the corresponding author on reasonable request.

## Abbreviations

FAI: Femoroacetabular impingement
SSI: Subspine impingement
AIIS: Anterior inferior iliac spine
3D-CT: Three-dimensional computerized tomography
MRI: Magnetic resonance imaging
LCEA: Lateral center-edge angle
ROM: Range of motion
